# Crosstalk with Jasmonic Acid Integrates Multiple Responses in Plant Development

**DOI:** 10.3390/ijms21010305

**Published:** 2020-01-02

**Authors:** Geupil Jang, Youngdae Yoon, Yang Do Choi

**Affiliations:** 1 School of Biological Sciences and Technology, Chonnam National University, Gwangju 61186, Korea; yk3@jnu.ac.kr; 2 Department of Environmental Health Science, Konkuk University, Seoul 05029, Korea; yyoon21@gmail.com; 3 The National Academy of Sciences, Seoul 06579, Korea

**Keywords:** jasmonic acid, crosstalk, gibberellic acid, cytokinin, auxin

## Abstract

To date, extensive studies have identified many classes of hormones in plants and revealed the specific, nonredundant signaling pathways for each hormone. However, plant hormone functions largely overlap in many aspects of plant development and environmental responses, suggesting that studying the crosstalk among plant hormones is key to understanding hormonal responses in plants. The phytohormone jasmonic acid (JA) is deeply involved in the regulation of plant responses to biotic and abiotic stresses. In addition, a growing number of studies suggest that JA plays an essential role in the modulation of plant growth and development under stress conditions, and crosstalk between JA and other phytohormones involved in growth and development, such as gibberellic acid (GA), cytokinin, and auxin modulate various developmental processes. This review summarizes recent findings of JA crosstalk in the modulation of plant growth and development, focusing on JA–GA, JA–cytokinin, and JA–auxin crosstalk. The molecular mechanisms underlying this crosstalk are also discussed.

## 1. Introduction

Plant growth and physiology are regulated by endogenous processes and environmental signals; phytohormones govern these processes by controlling transcriptional and translational networks. Jasmonates, including jasmonic acid (JA) and its derivatives, were initially isolated as a methyl ester form of JA in *Jasminum* *grandiflorum*. JA is classified as a cyclopentane fatty acid and is biosynthesized from linolenic acid, a major fatty acid of membranes in plant cells. Details of the JA biosynthetic pathway have been well reviewed [1,2]. Briefly, JA biosynthesis is regulated by enzymes such as lipoxygenase, allene oxide synthase, and allene oxide cyclase, which mediate the octadecanoid pathway. The free acid JA can be further metabolized into methyl jasmonate or the JA-isoleucine conjugate (JA-Ile) via the activity of jasmonate methyl transferase and jasmonate-amido synthetase, respectively. In response to environmental signals, the expression of the genes involved in JA metabolism is dynamically regulated, leading to changes in endogenous JA levels and stress responses, supporting the view that JA is a key hormone mediating plant responses to environmental stresses [3].

Early studies on JA showed that JA treatment rapidly and dynamically regulates genes involved in plant defense, suggesting the existence of a JA-specific signaling pathway and the integral role of JA in regulating gene expression networks [4,5]. In 1994, *Arabidopsis thaliana coronatine insensitive 1* (*coi1*) mutants, in which the JA response is blocked, were identified [6] and a series of studies on *coi1* extended our understanding of the JA signaling pathway. *COI1* encodes an F-box protein that acts as the JA receptor and functions in E3-ubiquitin ligase-mediated proteolysis of target proteins [7,8,9] such as the JASMONATE ZIM-DOMAIN (JAZ) proteins. Further identification of JA signaling components, including JA-responsive MYC transcription factors, revealed a JA signaling pathway that includes JA perception and JA-dependent gene regulation. Briefly, the expression of JA-dependent genes and activation of the JA response are inhibited in plant cells with low JA levels. In these cells, the MYC2 transcription factors, which are responsible for the expression of JA-responsive genes, stay inactive through the direct interaction with JAZ proteins, which are JA signaling repressors. JAZ proteins contain two domains, ZIM and Jas, and these domains mediate the interaction of JAZs with other proteins. The ZIM domain is responsible for its dimerization and interaction with NINJA, which connects the transcriptional suppressor TOPLESS to JA signaling, and the Jas domain mediates the JAZ–COI1 interaction [10,11].

When JA biosynthesis is activated in response to endogenous or environmental signals, and JA, especially JA-Ile, accumulates in cells, JA-Ile activates JA signaling through interaction with the COI1 receptor. This direct interaction induces proteolysis of the JAZ proteins and activates the expression of JA-responsive genes by releasing the MYC2 transcription factor from the JAZ–MYC2 complex [8]. Unlike the JAZ repressors, the MYC2 transcription factor activates the transcription of JA-responsive genes and promotes the JA response. As JAZs and MYC2 are key factors in plant growth and development as positive and negative regulators, respectively, they may mediate JA-dependent growth inhibition under stress conditions [12,13,14].

Plant hormones have their own specific biosynthetic and signaling pathways, but their roles in plant development and physiology overlap. This suggests that plant hormones modulate plant growth and physiology through interactions with other hormones, and the extensive interplay between auxin and cytokinin in the regulation of all aspects of plant growth and development supports this idea [15,16]. JA mediates the plant response to biotic and abiotic stresses through interaction with salicylic acid, ethylene, and abscisic acid (ABA), and details of this crosstalk and its underlying molecular mechanisms have been well reported in previous studies [3,17,18,19]. JA also modulates plant development, such as root, stamen, hypocotyl, chloroplast, and xylem development, and increasing evidence suggests that JA-dependent modulation of plant growth and development largely depends on the interaction of JA with other phytohormones such as gibberellins (GAs), cytokinin, and auxin that govern endogenous developmental programs. Many studies have revealed that the crosstalk between phytohormones is mediated through regulatory proteins controlling phytohormone metabolic and signaling pathways [3,20]. This review briefly describes the metabolism and signaling pathways of the phytohormones GA, cytokinin, and auxin that interact with JA in the modulation of plant growth and development, and recent findings on JA crosstalk, focusing on the JA–GA, JA–cytokinin, and JA–auxin interactions. The molecular mechanisms underlying the JA–GA, JA–cytokinin, and JA–auxin interactions are also discussed in this review.

## 2. The JA–GA Interaction

### 2.1. GA Metabolism and Signaling Pathway

GAs regulate plant growth and development, such as stem elongation, seed germination, leaf expansion, root development, and stamen and flower development [21]. Due to the essential role of GAs in plant growth, the GA response affects plant growth and productivity [22], and many studies suggest that GA is fundamental to stress-related growth inhibition through interactions with stress-response hormones [23,24,25,26,27,28,29,30].

GAs are a large class of tetracyclic diterpenoid compounds, and approximately 136 forms have been identified in higher plants and fungi. However, only a few of them, including GA_1_, GA_3_, GA_4_, and GA_7_, are biologically active, while other GAs are intermediate forms in the GA biosynthetic process or inactive forms of GAs. Therefore, GA metabolism, including its biosynthesis, is integral to GA homeostasis and the GA response in plants [31,32]. The biosynthetic pathway of GAs includes the biosynthesis of *ent*-kaurene, the conversion of *ent*-kaurene to GA_12_, and the formation of C_20_- and C_19_-GAs in the cytosol, and three different classes of enzymes, terpene synthases, cytochrome P450 monooxygenases, and 2-oxoglutarate-dependent dioxygenases, mediate this process [20,33,34]. Further metabolic processes are required for the formation of active GAs and the deactivation of bioactive GAs, and GA 20-oxidase, GA 3-oxidase, and GA 2-oxidase mediate these metabolic process [35,36,37,38].

GA signaling is another key step controlling the transcription of GA-dependent genes and the regulation of the GA response, and, similar to other plant hormones such as JA, auxin, and strigolactone, the GA signaling process is based on E3 ubiquitin ligase-mediated proteolysis of DELLAs. The Arabidopsis genome encodes five DELLAs, including REPRESSOR OF GA1-3 (RGA), which functions as an intracellular negative regulator of GA signaling [39,40]. In Arabidopsis, direct interaction between GAs and the GA INSENSITIVE DWARF1 (GID1) receptor induces the interaction between GID1 and DELLAs, and provokes the degradation of DELLAs through E3 ubiquitin ligase-mediated ubiquitinylation and 26S proteasome-mediated proteolysis [39,41,42]. The proteolysis of DELLAs leads to the release of GA-responsive transcription factors such as PHYTOCHROME INTERACTING FACTORS (PIFs) in Arabidopsis and PIF-LIKE (PIL) proteins in rice (*Oryza sativa*), and triggers the transcription of GA-responsive genes and the GA response [43,44,45]. The finding that *RGA*-overexpressing plants displayed a reduced GA response while mutants lacking *RGA* expression showed an enhanced GA response indicates a crucial role of DELLAs in GA signaling pathways [46,47,48].

### 2.2. The JA–GA Interaction and Its Underlying Molecular Mechanism

Environmental stresses strongly affect plant growth. To survive under stress conditions, plants activate defense programs and suppress developmental programs, leading to growth inhibition. By contrast, in the proper conditions for growth, plants activate developmental programs while suppressing defense programs, leading to vigorous growth. This indicates that plants dynamically coordinate growth and defense strategies in response to environmental stresses. The essential role that GAs play in the regulation of plant growth suggests that GAs have key roles in this coordination, and the finding that environmental stresses, such as salinity, promote the accumulation of DELLAs but reduce endogenous levels of bioactive GAs supports this idea [24,25,26]. In addition, stress-induced growth reduction was attenuated in quadruple-*della* mutants, while plants with reduced GA levels, such as the GA biosynthesis *mutant ga1*-*3*, exhibited enhanced tolerance to salt stress [24]. These findings indicated that GA plays an essential role in the coordination of plant growth and defense, and further analysis of *della* mutant plants suggested that DELLAs are deeply involved in GA-dependent coordination process [49].

**Figure 1:**
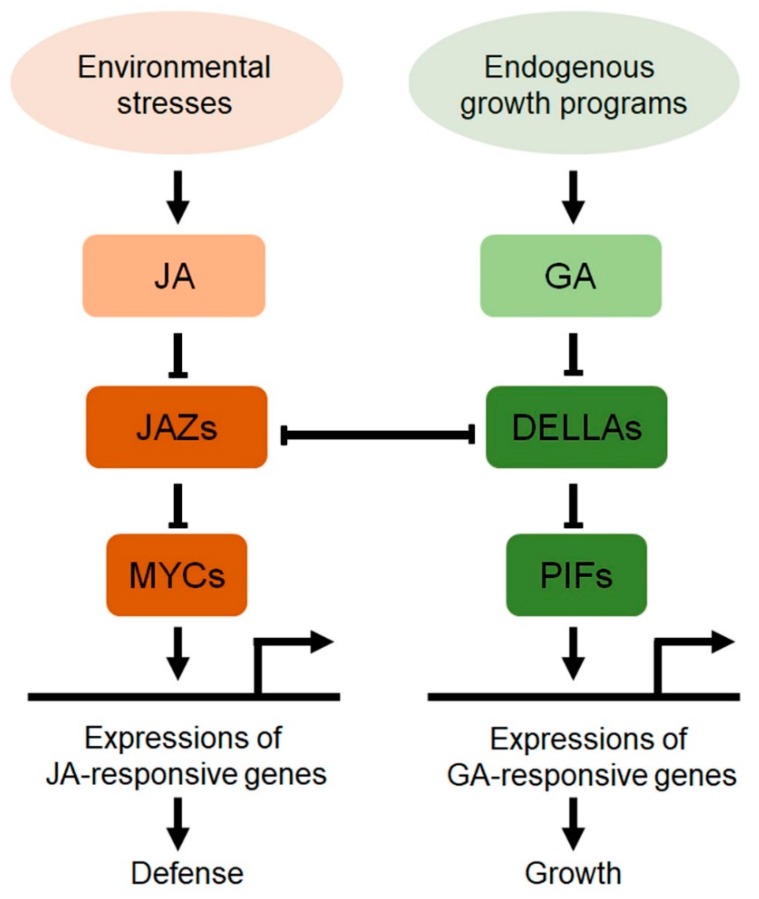
A schematic of crosstalk between jasmonic acid (JA) and gibberellic acid (GA) in coordination between plant growth and defense. JA and GA antagonistically interact to coordinate plant growth and defense, and the crosstalk is mediated by direct interaction between JA signaling repressors, JASMONATE ZIM-DOMAIN (JAZs), and GA signaling repressors, DELLAs. MYCs and PHYTOCHROME INTERACTING FACTORS (PIFs) indicates transcription factors responsible for transcription of JA-responsive and GA-responsive genes, respectively. The arrows and T bars indicate positive and negative regulation, respectively.

Many studies reported that developmental flexibility under stress conditions largely depends on the interplay between stress-related hormones and growth-related hormones, and increasing evidence indicates that JA and GA antagonistically interact to coordinate plant growth and defense [50,51,52]. Extensive studies on the JAZ JA signaling repressor proteins, and the DELLA GA signaling repressor proteins revealed that direct interaction between JAZs and DELLAs mediates the antagonistic interaction between JA and GA (Figure 1) [27,28]. In the “relief of repression” model, the JAZ–DELLA interaction attenuates the functions of JAZs and DELLAs as signaling repressors. For example, in GA-free conditions, DELLAs directly interact with JAZs and allow MYC2 to promote the JA response, while in the presence of GA, JAZs are released from the DELLA–JAZ complex by degradation of DELLAs, and the free JAZs attenuate the JA response through direct interact with MYC2. The model explained the DELLA-mediated upregulation of the JA response and the antagonistic interaction between JA and GA [27]. This model was supported by studies showing that JA promotes transcription of *RGA3*, and the JA-responsive MYC2 transcription factor directly binds to the promoter of *RGA3* [29].

A recent study using overexpression plants and knock-out mutants of *OsJAZ9* revealed that OsJAZ9 is a key JAZ protein that mediates the antagonistic interaction of JA and GA [21]. In this study, they identified OsJAZ9 proteins that directly interact with the rice DELLA protein SLENDER RICE 1 (SLR1), and demonstrated that the OsJAZ9–SLR1 interaction mediates the antagonistic interaction of JA and GA in rice by showing that overexpression of *OsJAZ9* promotes the GA response while knock-out of *OsJAZ9* reduces the GA response. Together, these data suggest that JA is an essential hormone that modulates plant growth under stress conditions, and its antagonistic interaction with GA mediates this process.

## 3. The JA–Cytokinin Interaction

### 3.1. Cytokinin Metabolism and Signaling

Cytokinin regulates the maintenance of stem cell identity and cell proliferation; therefore, cytokinin affects most aspects of plant growth and development [53]. The expression of genes involved in cytokinin responses is largely affected by the stress-response hormone JA or JA-dependent stress responses [54,55,56]. Furthermore, cytokinin-deficient mutant plants displayed increased tolerance to stresses, similar to transgenic plants with higher JA responses [57,58,59,60]. These studies suggested that the cytokinin response is integral to the JA-dependent stress response and growth modulation.

Most naturally occurring cytokinins are derivatives of isopentenyladenine, and zeatin is the ubiquitous form of cytokinins in higher plants [53,61]. Zeatin occurs as two isomers, *trans*-zeatin (*t*Z) and *cis*-zeatin (*c*Z); *t*Z is the active form of cytokinin in all plant species and *c*Z is less active than *t*Z [62,63]. Isopentenyl transferases (IPTs), and cytochrome P450 CYP735A1 and CYP735A2 mediate the production of *t*Z cytokinin [53]. The IPT-catalyzed reaction is the rate limiting step in cytokinin biosynthesis process, and the results showing that overexpression of *AtIPT1*, *3*, *4*, *5*, *7*, or *8* promoted cytokinin production and shoot growth support this [61,64,65]. The biological activity and homeostasis of cytokinins can be regulated by conjugation with glucose or amino acids, or by degradation. For example, glucosyl-conjugated cytokinins, which do not interact with cytokinin receptors, are inactive, and overexpression of cytokinin oxidase, which is responsible for cytokinin cleavage, reduces endogenous levels of cytokinins [66,67].

The cytokinin signaling pathway, which is composed of cytokinin receptors, histidine phosphotransfer proteins, and transcription factors, regulates cytokinin responses in plants. In Arabidopsis, three histidine kinases (AHK2, AHK3, and AHK4/WOODEN LEG) function as cytokinin receptors [53,68]. Direct interaction between cytokinins and the histidine kinase receptors activates the kinase activity of the receptors, leading to autophosphorylation on the conserved histidine residue. The phosphate is transferred to the histidine phosphotransfer proteins (AHPs) via the conserved aspartate residue of the receptors. In Arabidopsis, five genes encode AHPs that normally function as histidine phosphotransferases and one gene (*AHP6*) encodes a pseudo-AHP that negatively regulates cytokinin signaling. The AHPs activated by phosphorylation move into the nucleus and sequentially activate B-type ARABIDOPSIS RESPONSE REGULATOR (ARR) transcription factors responsible for the transcription of cytokinin-responsive genes [69,70]. Genes encoding components of the cytokinin signaling pathways, such as *AHKs*, *AHPs*, and *ARRs*, are affected by JA or environmental stresses such as drought, salt, and cold, suggesting that the cytokinin response is involved in plant stress responses [50,56,71,72].

### 3.2. The JA–Cytokinin Interaction and Its Underlying Molecular Mechanism

Previous studies have proposed that JA antagonistically interacts with cytokinin in various aspects of plant development. For example, JA inhibits cytokinin-induced soybean (*Glycine max*) callus growth [73], and nullifies the effect of cytokinin on chlorophyll development [74,75]. Furthermore, JA and cytokinin differently regulate the expression of the genes involved in the chlorophyll development, indicating the existence of an antagonistic interaction between JA and cytokinin. A recent study revealed that xylem differentiation is regulated by JA in Arabidopsis roots, and an antagonistic interaction between JA and cytokinin is fundamentally important for JA-dependent xylem development [50]. Xylem is responsible for water and nutrient transport and it develops from procambial/cambial cells, which are stem cells of the vascular system [76,77]. In Arabidopsis roots, cytokinin maintains stem cell identity and functions as a negative regulator of xylem differentiation. The role of cytokinin in xylem differentiation was demonstrated by showing that exogenous cytokinin treatment inhibits xylem development, and the *wooden leg* mutants with defects in cytokinin signaling strongly exhibit an all-xylem phenotype and lack procambial cells in their roots. Additionally, mutants that lack transcription of Type-B *ARRs*, such as *ARR1*, *ARR10*, and *ARR12*, or transgenic plants overexpressing *AHP6*, a negative regulator of cytokinin signaling, form extra xylem [50,78]. Similar to the cytokinin signaling mutants, the wild-type plants or JA-deficient *OPDA reductase 3* (*opr3*) mutants treated with exogenous JA showed an extra xylem phenotype, whereas JA signaling mutants, such as *coi1* and *jasmonate resistant 1* (*jar1*), did not [50]. Together with the results that JA suppresses the procambium-specific cytokinin response, and that the effect of JA on extra xylem formation is nullified by cytokinin, suggest that the stress hormone JA antagonistically interacts with cytokinin in xylem development in Arabidopsis roots.

**Figure 2:**
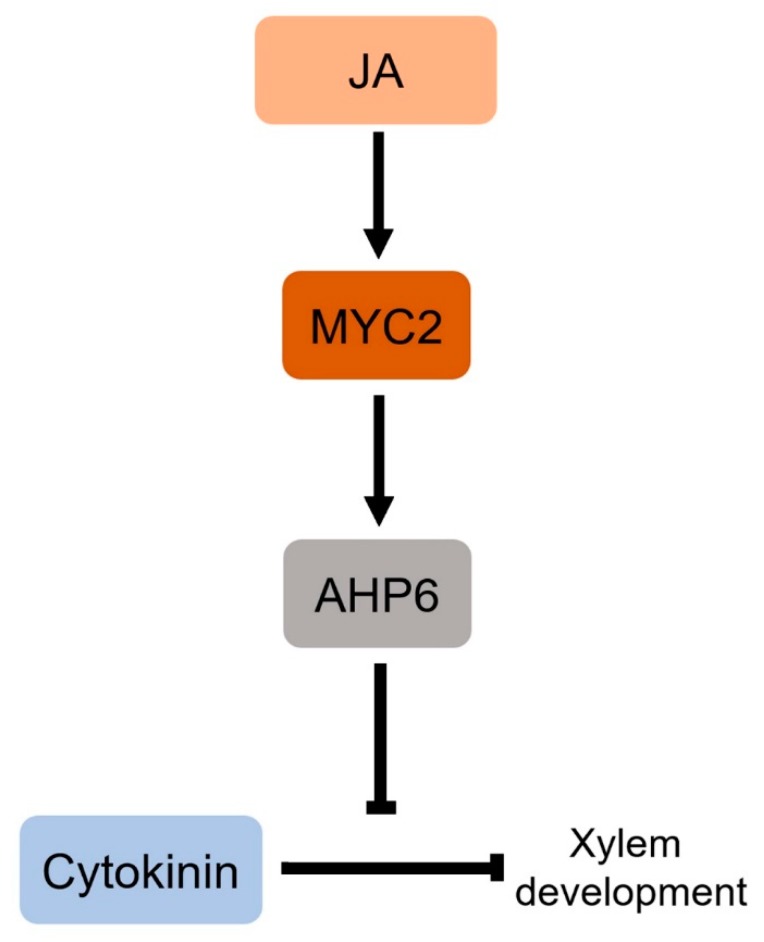
A schematic of crosstalk between JA and cytokinin in xylem development. JA antagonistically interacts with cytokinin in xylem development and the JA-responsive MYC2 transcription factor mediates this process. MYC2 negatively regulates cytokinin response by promoting expression of *AHP6*, a cytokinin signaling inhibitor. The arrows and T bars indicate positive and negative regulation, respectively.

These findings were supported by the results that JA reduces the expression of the cytokinin-responsive *PIN-FORMED* 7 (*PIN7*) gene, which is responsible for xylem development, and the finding that drought stress induces the formation of extra xylem in Arabidopsis roots further supported this idea [50,79]. Furthermore, *myc2* mutant did not form extra xylem in response to exogenous JA, and the expression of *AHP6*, encoding a cytokinin signaling inhibitor, was reduced in *myc2* mutant, suggesting that the JA-responsive MYC2 transcription factor mediates this process by promoting *AHP6* expression (Figure 2). It is likely that an antagonistic interaction between JA and cytokinin is also involved in the regulation of JA-dependent stress responses. A recent study by Nitschke et al. (2016) showed that plants with reduced cytokinin levels or defective cytokinin signaling exhibited a JA-dependent cell death phenotype in response to circadian stress, unlike wild-type plants [80], suggesting that JA and cytokinin antagonistically interact in the plant response to circadian stress.

Despite many studies supporting an antagonistic interaction between JA and cytokinin in modulation of plant development and physiology, the molecular mechanisms underlying the JA–GA crosstalk remain largely unknown. Regulation of *AHP6* expression by MYC2 transcription suggests that components of JA and cytokinin signaling pathways might mediate the interaction between JA and cytokinin. However, the observation that cytokinin levels were affected by stress conditions also suggests that regulation of JA and cytokinin metabolism might also be involved in the JA–cytokinin interaction [60,81,82].

## 4. The JA–Auxin Interaction

### 4.1. Auxin Metabolism and Signaling

Auxin has essential functions in cell fate determination and cell division, thus mediating most aspects of plant growth and development [83]. Indole-3-acetic acid (IAA) is the predominant form of natural auxins in plants. IAA can be produced through tryptophan-dependent and -independent pathways, and the tryptophan-dependent pathway is currently the best understood auxin biosynthetic pathway in plants [84,85]. The tryptophan-dependent pathway is mediated by tryptophan aminotransferase (TAA), and the flavin monooxygenase YUCCA (YUC). TAA and YUC are responsible for the conversion of tryptophan to indole-3-pyruvate (IPA) and the conversion of IPA to IAA, respectively. Similar to other plant hormones, IAA can be deactivated by conjugation with amino acids or sugars, and by oxidation [86].

Auxin-mediated regulation of gene expression is crucial for auxin-dependent regulation of plant growth and development, and this process is regulated by the auxin signaling pathway. Similar to JA and GA, auxin signaling is based on E3 ubiquitin ligase-mediated proteolysis of signaling repressor proteins [87]. AUXIN RESPONSE FACTORS (ARFs) are transcription factors responsible for the transcription of auxin-responsive genes, and they regulate the transcription of auxin-responsive genes by directly binding auxin responsive elements through their B3-like DNA binding motif. The transcriptional activity of ARFs depends on the interaction with the auxin signaling repressor Aux/IAAs, and degradation of Aux/IAAs induces the release of ARFs with transcriptional activity and activates the transcription of auxin-responsive genes. The degradation of Aux/IAAs is provoked by the SCF^TIR1^ E3 ubiquitin ligase, and the direct interaction between auxin and the TIR1 auxin receptor enhances the physical association between TIR1 and Aux/IAAs and sequential ubiquitination of Aux/IAAs [88].

### 4.2. Interaction of JA and Auxin and the Underlying Molecular Mechanism

The interaction of JA and auxin in plant development and physiology plays a role in processes such as cell elongation, tendril coiling, and the production of secondary metabolites [89,90], but this interaction has not been elucidated at the molecular level. The identification of genes involved in JA and auxin metabolism and signaling pathways have revealed that JA and auxin interact to modulate plant development.

**Figure 3:**
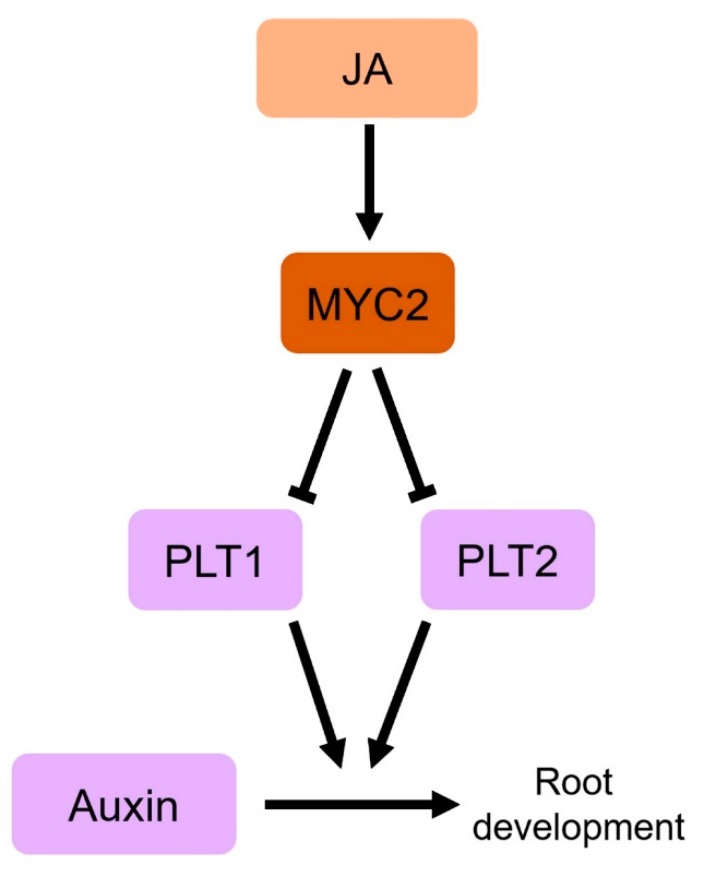
A schematic of crosstalk between JA and auxin in root development. PLT1 and 2 transcription factors are key regulators of root development downstream auxin. JA inhibits root growth, and MYC2 transcription factor mediates this development process by reducing expression of *PLT1* and *2*. The arrows and T bars indicate positive and negative regulation, respectively.

The interaction between JA and auxin has been well demonstrated in the regulation of root development. JA inhibits apical growth of roots; JA-treated wild-type plants form much shorter roots than untreated wild-type plants, while mutant plants with defects in JA signaling form similar roots in length to the roots of wild-type plants even in JA-treated conditions [50]. By contrast, auxin is essential for root growth and auxin deficiency or signaling mutants, such as (*trp2-12*) and *auxin resistant 3* (*arx3-1*), develop very short roots compared to wild-type plants [91,92]. This suggests that JA-induced inhibition of root growth might be mediated by an interaction with auxin, and a study by Chen et al. (2011) demonstrated this [93]. In the study, they showed that the JA-mediated inhibition of root growth is caused by a reduction of root meristem activity, and exogenous JA treatment suppresses the expression of the auxin-responsive transcription factors *PLETHORA*s (*PLTs*), which are responsible for maintenance of the stem cell niche and cell proliferation [94]. However, the expression levels of *PLTs* was not suppressed in JA-signaling mutants, such as *coi1-1* and *myc2*, suggesting that COI1-dependent JA signaling mediates the JA-induced root phenotype, and the MYC2 transcription factor suppresses the expression of *PLTs*. Together with the result that MYC2 directly binds to the promoters of *PLTs*, indicate that JA-responsive MYC2 mediates JA-induced inhibition of root growth by directly repressing the expression of auxin-responsive *PLTs* (Figure 3), and suggest that JA and auxin antagonistically interact in the regulation of apical root growth.

The JA–auxin interaction is involved in various aspects of plant development as well as root development. The development of floral organs, such as petals and stamens, is coordinated as flowers mature, and a study by Reeves et al. (2012) showed that an interaction between auxin-responsive transcription factors and JA biosynthesis modulates this process [95]. The R2R3 MYB transcription factors MYB21 and MYB24 are key regulators of petal and stamen growth, and the auxin-responsive transcription factors ARF6 and ARF8 regulate the expression of JA-responsive *MYB21* and *MYB24* by controlling JA biosynthesis, indicating that auxin interacts with JA to regulate the development of floral organs [95,96]. The JA-auxin interaction was also observed in the regulation of leaf senescence.

JA plays an essential role as a positive regulator of leaf senescence. JAZ7 suppresses dark-induced leaf senescence, while MYCs, including MYC2, promote senescence by activating the expression of senescence-associated genes and chlorophyll degradation-related genes, indicating that JA activates leaf senescence through a COI1-dependent JA signaling pathway [97,98,99]. In JA-dependent leaf senescence, the JA signaling repressors JAZ4 and JAZ8 function as negative regulators while the auxin signaling repressor IAA29 functions as a positive regulator. In JA-dependent leaf senescence, WRKY57 is another negative regulator that negatively affects the expression of senescence-associated genes. More importantly, WRKY57 interacts with JAZ4/8 and IAA29. These results suggest that competition between the WRKY57–JAZ4/8 and WRKY57–IAA29 interactions mediates JA-dependent leaf senescence, suggesting that an antagonistic interaction of JA and auxin is involved in leaf senescence [100].

## 5. Complexity of JA Crosstalk

Hormonal interactions are a critical component of plant growth and physiology [3]. This review described the role of JA crosstalk with other phytohormones in the modulation of plant growth and development, focusing on JA–GA, JA–cytokinin, and JA–auxin interactions and the molecular mechanisms underlying these processes. JA interacts with most plant hormones, and as shown in previous studies, JA extensively interacts with salicylic acid to modulate plant defenses against pathogen attacks. The interaction between JA, which mediates disease resistance to necrotrophic pathogens, and salicylic acid, which mediates broad-spectrum resistance to biotrophic pathogens, allows plants to establish an efficient defense system against a variety of pathogen attacks, and ethylene is also involved in this process [17,101]. In addition, ABA interacts with JA to regulate cellular metabolic processes, and ABA receptor PYRABACTIN RESISTANCE1-Like proteins with the ability to interact with JAZs mediate this process by modulating JA signaling [102].

Brassinosteroids (BR) mediate various aspects of plant growth and development and modulate JA signaling and JA-dependent growth inhibition. For example, *DWARF4* encodes a key enzyme responsible for BR biosynthesis and a leaky mutation of *DWARF4* restored JA sensitivity in the *coi1* mutant background and showed JA hypersensitivity in the wild-type background. Furthermore, expression of *DWARF4* was downregulated by JA in a COI1-dependent manner, and exogenous BR treatment attenuated the effects of JA on root growth inhibition [103]. These results indicate that a BR–JA interaction is involved in the modulation of JA signaling.

As described in this review, JA interacts with a variety of hormones involved in growth regulation, such as GA, cytokinin, auxin, and BR, to modulate plant growth and development, and the nature of the interaction is generally antagonistic. These interactions may help optimize plant growth and development under stress conditions. However, the nature of the interaction appears to differ depending on the type of cell and tissue. For example, JA and GA antagonistically interact in stem elongation, while they interact synergistically in stamen development [21,104]. JA antagonistically interacts with auxin to modulate apical growth of roots, but synergistically to promote lateral root growth [93,105].

## 6. Future Perspectives

Identification and characterization of the components involved in plant hormone metabolism and signaling have provided important clues to understand the hormonal interactions underlying the regulation of plant growth and physiology in response to endogenous and exogenous signals. JA is a key hormone that mediates the plant response to biotic and abiotic stresses, and is deeply involved in stress-induced modulation of plant growth and development. Increasing evidence indicates that JA-dependent growth regulation largely depends on the crosstalk of JA with other growth-related hormones such as auxin, cytokinin, GA, and BR. Although some of the molecular mechanisms underlying these processes have been revealed, including protein–protein interactions between hormone signaling components, many of the questions about the complexity and dynamics of hormonal interactions still remain unanswered. Further molecular and genetic studies will expand our understanding of the mechanisms underlying JA crosstalk in the modulation of plant growth and development under stress conditions.
